# Fear of Falling, Balance Disturbances, and Health-Related Quality of Life in Post-Stroke Inpatients: A Preliminary Cross-Sectional Study

**DOI:** 10.3390/jcm15051749

**Published:** 2026-02-25

**Authors:** Kacper Krysiak, Maciej Miś, Marcin Miś, Adam Druszcz, Małgorzata Paprocka-Borowicz, Joanna Rosińczuk, Robert Dymarek

**Affiliations:** 1 University Centre of Rehabilitation and Physiotherapy, Wroclaw Medical University, 55-355 Wroclaw, Poland; kacperk1554@wp.pl; 2 Department of Neurosurgery, University Centre of Neurology and Neurosurgery, Wroclaw Medical University, 50-556 Wroclaw, Poland; mis.neurochirurg@gmail.com; 3 Department of Radiology, Interventional Radiology and Neuroradiology, Wroclaw Medical University, 50-556 Wroclaw, Poland; marcin.mis@gmail.com; 4 Department of Neurosurgery, Provincial Specialist Hospital in Legnica, 59-220 Legnica, Poland; dr.druszcz@gmail.com; 5 Department of Neurological Rehabilitation, Regional Specialist Hospital in Wroclaw, 51-128 Wroclaw, Poland; malgorzata.paprocka-borowicz@wssk.wroc.pl; 6 Division of Internal Medicine Nursing, Department of Nursing, Wroclaw Medical University, 51-618 Wroclaw, Poland; joanna.rosinczuk@umw.edu.pl; 7 Division of Clinical Physiotherapy and Rehabilitation, University Centre of Rehabilitation and Physiotherapy, Wroclaw Medical University, 55-355 Wroclaw, Poland

**Keywords:** stroke, balance, fall risk, rehabilitation, functional independence

## Abstract

**Background/Objectives**: Fear of falling is highly prevalent after stroke and may interact with mobility limitations, neurological deficits, and reduced quality of life. However, few studies have examined these dimensions together during inpatient rehabilitation. This study aimed to assess fear of falling, balance and gait performance, and HRQoL in adults undergoing post-stroke inpatient rehabilitation, and to examine the interrelationships among psychological, functional, and clinical measures to support multidimensional assessment strategies. **Methods**: This cross-sectional study included 39 patients (51.28% women, 71.79% after ischemic stroke) undergoing post-stroke inpatient rehabilitation. The main assessments included the Falls Efficacy Scale-International (FES-I), the Tinetti Test (TT), and the Short Form-36 (SF-36). **Results**: High fear of falling was reported by 61.5% of participants, while a substantial proportion (35.9%) demonstrated moderate-to-high fall risk on the TT, despite the majority being classified as low risk. SF-36 domain scores indicated moderate HRQoL with substantial interindividual variability across dimensions. Strong correlations were found between SF-36 and FES-I (rs = 0.76, *p* < 0.001), TT (rs = −0.78, *p* < 0.001), Barthel Index (rs = −0.71, *p* < 0.001), and NIHSS (rs = 0.65, *p* < 0.001). Patients with greater neurological impairment and lower independence demonstrated worse HRQoL and higher fear of falling. Also, a statistically significant (*p* < 0.001) strong negative correlation (rs = −0.89) was found between the TT and the FES-I scores, indicating that higher fear of falling was associated with poorer mobility. **Conclusions**: Fear of falling, impaired balance, and reduced HRQoL are highly prevalent and strongly interconnected among post-stroke inpatients. These findings highlight the need for early multidimensional assessment and integrated interventions targeting both physical performance and psychological factors. Larger longitudinal studies are required to clarify causal pathways and optimize rehabilitation strategies.

## 1. Introduction

Stroke is one of the leading causes of long-term disability worldwide, frequently resulting in persistent impairments in mobility, balance, and physical confidence [1]. Beyond the direct motor consequences, many stroke survivors develop fear of falling (FoF)—a psychological construct characterized by reduced self-confidence in maintaining balance during daily activities and excessive concern about falling [2,3]. FoF is clinically important because it is not restricted to individuals who have previously fallen; it may arise early after stroke and persist even in patients with relatively good motor recovery [4].

Growing evidence indicates that elevated FoF contributes to activity avoidance, reduced mobility, functional decline and diminished quality of life, thereby reinforcing a self-perpetuating “fear-avoidance cycle” [5]. This phenomenon has been observed even in individuals with relatively mild motor deficits, suggesting that FoF reflects both psychological and sensorimotor factors.

Studies show that higher FoF is associated with slower gait speed, impaired dynamic balance, reduced community ambulation, and decreased participation in daily and social activities [6,7]. In addition, FoF has been linked to lower levels of physical activity and weakened postural control, which may further increase fall risk and exacerbate disability over time [8]. Consequently, elevated FoF has a substantial impact on health-related quality of life, affecting physical functioning, emotional wellbeing and social participation [9].

Studies in post-stroke populations have reported that higher FoF is associated with poorer gait performance, impaired balance control, decreased walking speed, and increased sedentary behavior [10]. However, most of these investigations have been conducted in Western European or Asian populations, and there remains a notable scarcity of data from Central and Eastern Europe, including Poland [11].

Moreover, although previous studies have examined selected associations, such as fear of falling in relation to gait parameters or balance confidence and quality of life [4,12,13], the majority of existing research has focused on single-domain or paired outcomes rather than integrating neurological severity, functional mobility, fear of falling, and health-related quality of life within a single analytical framework. To date, there remains limited evidence combining standardized measures such as the Falls Efficacy Scale-International, Tinetti Test, and SF-36 simultaneously, particularly in inpatient post-stroke rehabilitation settings in Central and Eastern Europe, including Poland.

Given the functional and psychological relevance of FoF after stroke, and the limited number of multidimensional analyses in this regional context, there is a clear need for preliminary exploratory studies to investigate its associations with mobility, balance performance and health-related quality of life. Using a cross-sectional design with validated instruments such as the Falls Efficacy Scale-International (FES-I), the Tinetti Test (TT), and a generic health-related quality-of-life questionnaire (e.g., SF-36), may provide a comprehensive picture of fear-related functional limitations in post-stroke patients. Understanding these multidimensional relationships is clinically relevant, as it may guide early identification of high-risk patients and inform holistic rehabilitation planning that addresses both physical function and psychological barriers to recovery.

Therefore, the general aim of this preliminary cross-sectional study was not only to quantify fear of falling in post-stroke inpatients but also to examine its interrelationships with balance and gait performance, neurological impairment, functional independence, and health-related quality of life within a real-world inpatient rehabilitation context. By identifying how these physical and psychological domains interact early during rehabilitation, this study seeks to support the clinical need for integrated, multidimensional assessment and intervention strategies rather than isolated outcome monitoring.

## 2. Materials and Methods

### 2.1. Study Design and Settings

This study was designed as a preliminary cross-sectional observational study. The study was conducted at the Gorzno Rehabilitation Center (Poland), a specialized inpatient rehabilitation facility providing post-stroke rehabilitation services. Patient recruitment was carried out consecutively over a defined inclusion period from September 2024 to March 2025. All eligible patients admitted during this period who met the inclusion criteria and provided informed consent were considered for participation. All assessments were performed during the patients’ rehabilitation stay under standardized clinical conditions. Data collection included functional measures, gait and balance evaluation, fear of falling assessment, and health-related quality of life profiling. The study adhered to the STROBE reporting recommendations for observational studies [14].

### 2.2. Ethical Considerations

The study was conducted in accordance with the Declaration of Helsinki and approved by the Independent Bioethics Committee of Wroclaw Medical University, Poland (no. KB–36/2024, date 29 February 2024). All participants provided written informed consent prior to enrollment and were informed of the study aims, procedures and their right to withdraw at any time without consequences for the therapeutic process.

### 2.3. Qualification Criteria

Inclusion criteria were: diagnosis of ischemic or hemorrhagic stroke confirmed by neuroimaging; age ≥18 years; ability to maintain a sitting position and follow verbal instructions; medically stable status allowing safe performance of functional tests; informed consent to participate. Exclusion criteria comprised: severe cognitive impairment preventing comprehension of test procedures, assessed by the attending neurologist through routine clinical evaluation supported by standardized cognitive screening (Mini-Mental State Examination or Montreal Cognitive Assessment, where available), with exclusion typically applied to patients presenting scores indicating severe impairment; severe aphasia preventing task execution, determined by neurological examination and functional communication assessment; comorbid neurological or orthopedic conditions significantly impairing gait (e.g., Parkinson’s disease, acute lower-limb injury); and cardiorespiratory instability or contraindications to mobility testing. Participant inclusion criteria were defined collaboratively with expert neurologists, neurosurgeons, and rehabilitation physicians, ensuring that the selection process reflected evidence-based practice and the clinical complexity of post-stroke rehabilitation.

### 2.4. Study Participants

Participants were adult patients undergoing post-stroke inpatient rehabilitation. Participants were recruited across different stages of post-stroke recovery, ranging from the early sub-acute phase to chronic stages, reflecting the heterogeneous population typically admitted to inpatient rehabilitation. All were clinically stable and capable of completing functional assessments with verbal instruction. Demographic and clinical data were collected through structured interviews and medical record review. Stroke subtype (ischemic or hemorrhagic), time since stroke onset, comorbidities, and initial neurological status were documented.

During the study period, 52 consecutive post-stroke inpatients were screened for eligibility. Thirteen patients were excluded due to severe cognitive impairment (n = 5), inability to maintain a sitting position or follow verbal instructions (n = 6), or medical contraindications to functional testing (n = 2). Ultimately, 39 patients met the inclusion criteria and were enrolled in the study.

### 2.5. Rehabilitation Program

Patients were admitted to the inpatient rehabilitation program at varying stages following stroke, most commonly within the early sub-acute period (approximately 1–3 months post-event), although individuals in later recovery phases were also included. The rehabilitation program at the Gorzno Rehabilitation Center follows a multidisciplinary model and typically includes daily physiotherapy focused on gait training, balance exercises, strength and mobility improvement, and task-oriented functional training, complemented by occupational therapy targeting activities of daily living, and, when indicated, speech and cognitive therapy. Rehabilitation was delivered by qualified rehabilitation physicians, physiotherapists, and therapists trained in established neurophysiological methods, including Proprioceptive Neuromuscular Facilitation (PNF) and the Bobath (Neurodevelopmental Treatment) concept. Therapy sessions were generally conducted five days per week, with individualized intensity based on patients’ functional status and medical condition.

### 2.6. Outcome Measures

All assessment instruments (including NIHSS [15] and BI [16]) were applied in their Polish-language versions that have undergone formal translation, cultural adaptation, and psychometric validation for use in Polish populations. These versions have demonstrated good reliability and validity in clinical and research settings in Poland.

#### 2.6.1. Fear of Falling

The Falls Efficacy Scale-International (FES-I) is a widely validated measure assessing concern about falling during everyday activities in older adults and people with neurological conditions, including stroke. The questionnaire consists of 16 items, each scored from 1 (“not at all concerned”) to 4 (“very concerned”), producing a total score from 16 to 64, with higher scores indicating greater fear of falling. The FES-I demonstrates excellent internal consistency (α > 0.90), strong test–retest reliability, and good construct validity across different clinical populations. It has been recommended as a standard outcome for assessing fear of falling due to its sensitivity to functional and psychological changes [17]. The Polish version of the FES-I has been culturally adapted and validated, showing high internal consistency and reliability in Polish clinical populations [18].

#### 2.6.2. Gait and Balance

The Tinetti Test (TT), also known as the Tinetti Performance-Oriented Mobility Assessment, is a comprehensive clinical tool used to assess balance and gait performance in older adults and individuals with neurological deficits. It consists of two subscales: Balance (0–16 points) and Gait (0–12 points). The total score ranges from 0 to 28, where lower scores reflect impaired mobility and a higher risk of falling. The TT is widely used in stroke rehabilitation due to its strong predictive validity for falls, moderate-to-high interrater reliability, and ability to detect functional deficits not captured by gait speed alone [19]. The Tinetti Test is a standard clinical tool for balance and gait assessment in Polish rehabilitation practice [20,21,22].

#### 2.6.3. Health-Related Quality of Life

The Short Form-36 (SF-36) is a widely used standardized questionnaire for assessing health-related quality of life (HRQoL) in clinical and research settings. It contains 36 items grouped into eight domains: Physical Functioning, Role Physical, Bodily Pain, General Health, Vitality, Social Functioning, Role Emotional, and Mental Health. Scores for each domain range from 0 to 100, with higher values indicating better perceived health. The SF-36 has been validated in stroke populations and is sensitive to functional, psychological and social consequences of post-stroke disability [23]. The SF-36 questionnaire was administered using its validated Polish version, which has undergone cultural adaptation and demonstrated good psychometric properties in Polish populations [24]. SF-36 outcomes were analyzed and reported primarily at the level of the eight validated domains. For descriptive purposes only, an overall SF-36 index was calculated as the arithmetic mean of the eight domain scores; this value does not represent a validated SF-36 summary measure and should not be interpreted as a standardized composite score such as the Physical or Mental Component Summary.

### 2.7. Sample Size

Given the exploratory and correlational character of this project, no a priori sample size calculation was performed, and the study was planned as a preliminary cross-sectional investigation including all consecutive eligible patients. After data collection, a post hoc power analysis was conducted using G*Power 3.1 (Heinrich Heine University, Düsseldorf, Germany). For correlational analyses (Spearman’s ρ), assuming a two-tailed α = 0.05, the obtained sample of n = 39 provided statistical power above 0.80 for medium effect sizes (ρ ≈ 0.40–0.50) and exceeding 0.95 for large effect sizes (ρ ≥ 0.60). As the primary associations observed in this study were in the medium-to-large range, the sample size was sufficient for detecting clinically meaningful relationships. The final sample size reflects the number of consecutive eligible patients admitted during the inclusion period and was determined by feasibility within a single-center inpatient rehabilitation setting rather than by formal a priori power calculation. Importantly, the adequacy of this cohort for correlational analyses is supported by prior post-stroke rehabilitation studies examining associations between fear of falling, mobility, functional independence, and quality of life, which have commonly employed sample sizes ranging from approximately 12 to 77 participants and reported meaningful correlations [12,13,25,26]. Given that the present study focused on identifying relationship patterns rather than on multivariable prediction or causal inference, the included sample size was considered appropriate for addressing the stated aims.

### 2.8. Statistical Analysis

Statistical analyses were performed using Statistica 14.0 (TIBCO Software Inc., Palo Alto, CA, USA). Continuous variables were assessed for normality using the Shapiro–Wilk test and are reported as mean ± standard deviation (SD) or median with interquartile range (IQR), depending on data distribution. Group comparisons were conducted using the Mann–Whitney U test for two-group analyses and the Kruskal-Wallis test for comparisons across three or more groups. When the Kruskal-Wallis test indicated significant differences, Dunn’s post hoc tests with adjusted *p*-values were applied to identify between-group contrasts. Categorical variables were analyzed using the chi-square test or Fisher’s exact test, as appropriate. To address the study objectives, differences in fear of falling severity (FES-I scores) across demographic (sex, place of residence) and clinical subgroups (stroke type, time since stroke) were examined using non-parametric group comparison tests. Associations between fear of falling (FES-I), balance and gait performance (TT), HRQoL (SF-36 domains), and continuous clinical variables, including neurological deficit severity (NIHSS) and functional independence (Barthel Index), were assessed using Spearman’s rank correlation coefficients. All statistical tests were two-tailed, and statistical significance was set at *p* < 0.05.

## 3. Results

### 3.1. Demographic Characteristics

The study included 39 post-stroke adults, with a balanced sex distribution (51.28% women, 48.72% men). The participants represented primarily older age groups: 38.46% were aged 70–79 years, 35.90% were aged 60–69 years, 17.95% were ≥80 years, and only 7.69% were under 60 years of age. Most participants were married (56.41%), followed by widowed individuals (28.21%), whereas only 5.13% were single. Regarding place of residence, 35.90% lived in towns with fewer than 50,000 inhabitants, 35.90% in larger cities (>50,000), and 28.21% in rural areas. Educational level was predominantly secondary education (87.18%), with only 10.26% reporting higher education and 2.56% primary education. These demographic characteristics are specified in Table 1.

### 3.2. Clinical Characteristics

Among participants, 71.79% had experienced an ischemic stroke, whereas 28.21% suffered a hemorrhagic stroke. Stroke lateralization was primarily right-sided (66.67%), while 48.72% reported left-hemisphere involvement (multiple indications allowed for some patients). The time since the most recent stroke varied: 43.59% were 1–3 months post-stroke, 23.08% were 3–6 months post-stroke, 20.51% exceeded 6 months, and 12.82% were within the first month after the event. Most patients had experienced one stroke (82.05%), while 15.38% had two strokes, and one patient (2.56%) had three strokes. Neurological deficits were common: 74.36% reported gait difficulties, 66.67% had balance or coordination impairments, 53.85% had left-sided paresis and 46.15% right-sided paresis, 20.51% experienced speech impairments, 12.82% had visual disorders, 7.69% experienced cognitive deficits, and 2.56% experienced auditory disturbances. One patient (2.56%) reported no neurological deficits. These clinical characteristics are specified in Table 2.

### 3.3. Fear of Falling (FES-I)

Fear of falling was prevalent in the study group. Based on FES-I cut-off scores: 12.82% reported no or minimal FoF, 25.64% had moderate FoF, and 61.54% reported high fear of falling. Mean FES-I scores were comparable across sex, stroke type, residence, and stroke chronicity groups (all *p* > 0.05). Full distributions are presented in Table 3.

### 3.4. Gait and Balance Performance (Tinetti Test)

Assessment using the Tinetti Test (TT) revealed that 17.95% of patients were at high fall risk (0–18 points), 17.95% were at moderate risk (19–23 points), and 64.10% were at low risk (24–28 points). TT scores did not significantly differ by sex (*p* = 0.725), stroke type (*p* = 0.121), place of residence (*p* = 0.605), or time since last stroke (*p* = 0.080). These findings indicate that functional mobility—which strongly contributes to fall risk—was reduced in a notable proportion of participants (Table 4).

### 3.5. Health-Related Quality of Life (SF-36)

The overall SF-36 domain scores indicated a moderate level of HRQoL, with substantial variability across individual dimensions. The highest mean scores were observed for Social Functioning (71.8 ± 25.9) and Bodily Pain (68.6 ± 23.9), suggesting relatively preserved social participation and pain-related functioning. In contrast, lower mean values were noted for General Health (60.8 ± 22.7) and Vitality (61.3 ± 24.6), reflecting moderate impairment in perceived overall health status and energy levels. The SF-36 total score, calculated as the mean of the eight domains, yielded a mean value of 65.4 ± 18.9, with a median of 66.9 (IQR: 55.4–78.6), confirming marked interindividual heterogeneity in overall HRQoL (Table 5). Descriptively, the global SF-36 index (mean of eight domains) showed similar values across sex, place of residence, stroke chronicity, and stroke type; therefore, HRQoL interpretation was based primarily on the validated domain scores.

### 3.6. Selected Comparisons

To address one of the main study objectives exploring whether demographic and clinical factors influence the severity of fear of falling, comparisons of FES-I and Tinetti Test outcomes were performed across key sociodemographic and clinical subgroups.

Across all sociodemographic and clinical subgroups: women had slightly higher FES-I scores and lower TT scores, but differences were not significant (Table 6); hemorrhagic stroke patients tended to have lower TT scores, though differences were not statistically significant (Table 7); place of residence did not differentiate levels of FoF or mobility (Table 8); time since stroke also did not significantly influence outcomes, and although the <1 month group showed higher FoF and lower TT performance, results were nonsignificant (Table 9).

### 3.7. Selected Correlations

**Figure 1:**
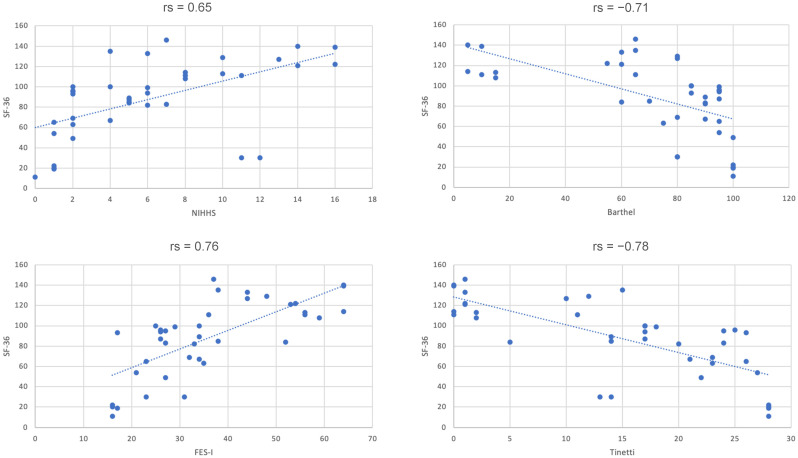
Correlations between key functional measures.

Strong and statistically significant associations were observed between health-related quality of life (SF-36) and key clinical measures (Table 10 and Figure 1). Higher SF-36 scores, indicating better perceived health status, were strongly correlated with lower fear of falling measured by FES-I (rs = 0.76, *p* < 0.001). Conversely, worse balance and gait performance on the Tinetti Test were associated with lower quality of life (rs = −0.78, *p* < 0.001), confirming that mobility limitations are closely linked to subjective well-being.

Neurological deficit severity also showed a moderately strong positive correlation with SF-36 (NIHSS: rs = 0.65, *p* < 0.001), suggesting that patients with more pronounced deficits reported poorer quality of life. Similarly, functional independence was significantly related to SF-36 (Barthel Index: rs = −0.71, *p* < 0.001), indicating that individuals with lower ADL performance perceived their health status as worse.

Overall, these findings demonstrate a coherent clinical profile, where diminished mobility, greater fear of falling, lower independence, and higher neurological impairment all contribute to worse health-related quality of life in post-stroke rehabilitation patients.

**Figure 2:**
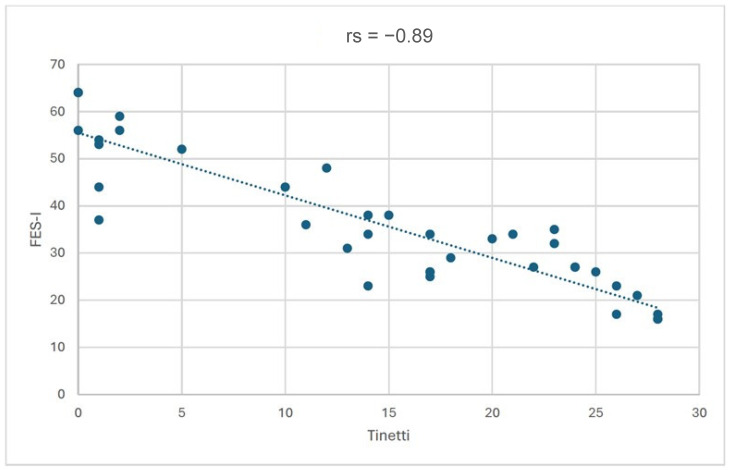
Correlation between FES-I and Tinetti scores.

In addition, a statistically significant (*p* < 0.001) strong negative correlation (rs = −0.89) was found between the Tinetti questionnaire score and the FES-I questionnaire score. It was observed that the lower the risk of falling, the lower the fear of falling (Figure 2).

## 4. Discussion

### 4.1. Summary and Interpretation of Main Findings

This cross-sectional study showed that post-stroke inpatients present a constellation of problems including high fear of falling, marked balance and gait impairments, and substantially reduced HRQoL. More than 60% of participants reported a high level of fear of falling on the FES-I although the majority of participants were classified as low fall risk according to the TT, a substantial proportion still demonstrated moderate-to-high risk, and high fear of falling was prevalent across the cohort. Strong correlations were observed between HRQoL and fear of falling, balance performance, functional independence, and neurological deficit, suggesting a tightly interlinked pattern of physical and psychological vulnerability. Importantly, the strong inverse association between fear of falling and objective balance and gait performance confirms that higher perceived fall-related concern is closely linked to reduced mobility, directly supporting the study’s primary objective. Notably, no significant differences in fear of falling, balance performance, or health-related quality of life were observed across key subgroups, including sex, stroke type, place of residence, and time since stroke. The consistently high levels of fear of falling across these categories suggest that this psychological burden is pervasive among post-stroke inpatients and not confined to specific demographic or clinical profiles. The rehabilitation-oriented recommendations proposed in this study are directly informed by the observed relationships between fear of falling, balance and gait performance, neurological impairment, functional independence, and health-related quality of life, rather than by isolated outcome measures

These results are consistent with contemporary evidence indicating that fear of falling is common after stroke and is closely related to both falls and broader health outcomes. A recent systematic review and meta-analysis by Pin et al. [27] showed a significant, albeit small, association between fear of falling and actual falls in both acute and chronic stroke populations, highlighting fear of falling as an independent clinical target rather than merely a consequence of previous falls. Structural equation modeling by Chen et al. [28] further demonstrated that in older stroke survivors, maladaptive fear of falling contributes to increased fall risk through its links with depression, reduced balance ability, fall history, and low physical activity, reinforcing the multifactorial nature of this problem.

The strong associations observed in our study between fear of falling, balance, and HRQoL align with cross-sectional and longitudinal data from stroke and broader cerebrovascular cohorts. Schmid et al. [29] reported that fear of falling is likely to slow overall recovery trajectories and restrict participation in clients with chronic stroke receiving occupational therapy, while Tahiraj et al. [2], analyzing European longitudinal data, found that a history of cerebrovascular disease is independently associated with higher fear of falling among middle-aged and older adults. Together with our findings, these studies underscore that fear of falling should be viewed as a key component of post-stroke disability rather than a secondary symptom.

### 4.2. Prevalence, Risk Profiles and Trajectories of Fear of Falling After Stroke

Our observation that a majority of inpatients experienced at least moderate fear of falling is in line with the wide prevalence range (32–83%) reported in the systematic review and meta-analysis by Xie et al. [10] That review identified female sex, impaired balance, lower mobility, use of walking aids and prior falls as the main correlates of fear of falling in stroke survivors—factors that are also prominent in our cohort (notably poor balance and gait).

Case–control data by Goh et al. [30] showed that persons with stroke are significantly more likely than matched controls to experience recurrent falls and fear of falling, and that fear of falling is predicted by functional ambulation level rather than by standard impairment measures. Hussain et al. [31] extended this evidence longitudinally, demonstrating that fear of falling at 6 months after stroke can be predicted already in the acute phase based on baseline mobility limitations and self-reported walking difficulties. Our findings of strong associations between FES-I, Tinetti scores, and ADL independence fit well within this predictive framework, suggesting that early identification of patients with combined mobility limitation and high fear of falling could enable targeted preventive interventions.

More recently, Yang et al. [32] used latent profile analysis to classify elderly stroke patients into high-, moderate-, and low-fear-of-falling subgroups and showed that demographic, sensory, motor, coping, and social support factors differentiated these profiles. Tian et al. [33], in a systematic review of contributing factors and interventions, further confirmed that female sex, assistive device use, balance dysfunction, limb impairment and limited functional mobility are central determinants of fear of falling, and highlighted combined cognitive-behavioral and exercise approaches as the most effective intervention strategies. The profile of our cohort, older adults with marked balance and gait disturbances and frequent use of assistive devices, corresponds closely to the high-risk groups described in those studies.

### 4.3. Relationship Between Fear of Falling, Falls and Quality of Life

Our data add to accumulating evidence that fear of falling and HRQoL are tightly interconnected after stroke. Chen et al. [28] reported that in older patients with stroke, fear of falling and falls are reciprocally related and jointly limit rehabilitation exercise, mobility and independence. Djurovic et al. [34] showed that falls in stroke patients are associated not only with neurological impairment and cerebrovascular lesion characteristics but also with mental status changes, behavioral adaptation, and difficulties accepting activity restrictions. In broader geriatric populations, large-scale analyses have demonstrated that fear of falling is associated with lower HRQoL, partly mediated by depression and activity restriction, and that this detrimental pathway is attenuated by higher physical activity levels [35].

These external findings mirror the pattern in our cohort, where higher fear of falling and lower balance performance correlated strongly with worse SF-36 scores. Our results thus support the notion that fear of falling is both a consequence and a driver of reduced activity and quality of life. The strong correlations between SF-36, FES-I and Tinetti scores are in line with previous studies indicating that balance confidence and fear of falling are closely linked to gait characteristics, cautious walking strategies and reduced participation after stroke [4].

An important finding of this study is the divergence between subjective fear of falling and objective balance and gait performance. While most patients were classified as low fall risk according to the Tinetti Test, more than 60% reported high fear of falling. This discrepancy highlights that fear of falling represents a distinct psychological construct that is not perfectly aligned with objective functional capacity. Similar patterns have been reported in previous stroke and geriatric populations, where elevated fear of falling persists despite relatively preserved mobility. This emphasizes that fear-related activity restriction and reduced confidence may occur independently of measurable balance deficits and should therefore be addressed as a separate therapeutic target.

### 4.4. Implications of Emerging Intervention Evidence

Although our study was observational, the pattern of associations aligns closely with emerging intervention literature. A systematic review and meta-analysis by Chiu et al. [36] showed that physical exercise programs, especially walking-focused interventions, significantly reduce fear of falling in individuals with stroke, with larger effects in those with poorer baseline balance. A more recent systematic review by Tian et al. [33] confirmed that combining exercise with cognitive–behavioral components yields the most robust improvements in fear of falling among stroke survivors.

Parallel lines of evidence suggest that balance-specific and technology-enhanced training (including virtual reality-based exercise) can improve balance, enhance confidence and lower fear of falling in chronic stroke and older adult populations [37]. These interventions may be particularly relevant for patients such as those in our cohort, who exhibit combined balance limitations, high fear of falling and reduced HRQoL. Integrating such approaches into inpatient and early post-discharge rehabilitation could therefore address not only physical performance but also the psychological aspects that undermine participation and quality of life.

In addition to exercise-based and cognitive-behavioral approaches, growing evidence highlights the role of environmental and footwear-related modifications as complementary strategies for improving balance, gait stability, and reducing fear of falling. Recent studies in stroke survivors and older adult populations have demonstrated that specific footwear characteristics, including enhanced sole stability, optimized grip, and supportive design, can positively influence postural control, gait performance, and perceived confidence during ambulation. For example, footwear interventions have been shown to improve balance parameters and reduce instability in individuals after stroke and in older adults at risk of falls, thereby contributing to lower fear of falling and improved functional mobility [38,39,40]. Incorporating appropriate footwear recommendations into rehabilitation and fall-prevention programs may therefore represent a simple yet effective adjunct to conventional therapeutic interventions.

### 4.5. Contribution of the Present Study

Within the existing literature, the main contribution of the present study lies in providing preliminary, real-world data from an inpatient post-stroke rehabilitation setting in Central and Eastern Europe, simultaneously examining neurological severity, functional independence, fear of falling, balance and gait performance, and health-related quality of life. Although limited by its exploratory design and single-center sample, the observed strong intercorrelations among these domains support the relevance of integrated multidimensional assessment in clinical practice, while underscoring the need for larger, confirmatory studies to more conclusively establish these relationships [10].

Our findings complement these reviews by demonstrating, in a real-world clinical setting, that fear of falling, balance impairments and reduced HRQoL are tightly interrelated and already clearly expressed during inpatient rehabilitation. This supports calls for early screening and comprehensive, multimodal interventions that simultaneously address motor performance, confidence, fear of falling and participation, rather than treating these dimensions separately.

### 4.6. Potential Study Limitations

Several limitations should be considered when interpreting these findings. First, the cross-sectional design precludes causal inferences regarding the relationships among fear of falling, mobility, neurological impairment and HRQoL; longitudinal studies are needed to track these dynamics over time. Second, the relatively small sample size limits the precision of correlation estimates and restricts the generalizability of the results. The observed associations should therefore be interpreted as exploratory and hypothesis-generating, warranting confirmation in larger, adequately powered multicenter studies. Third, reliance on self-reported tools such as SF-36 and FES-I introduces the possibility of response bias, despite their strong validation in stroke populations. Fourth, gait and balance were evaluated solely with the Tinetti Test; more advanced or instrumented measures could offer deeper biomechanical insight. Additionally, the inclusion criteria requiring the ability to maintain a sitting position and follow verbal instructions likely resulted in the exclusion of more severely impaired stroke patients. This may have introduced selection bias toward individuals with relatively better functional status and limits the generalizability of the findings to patients with profound neurological deficits or severe cognitive impairment. Detailed characteristics of excluded patients were not systematically collected and could therefore not be analyzed. Moreover, patients were included across varying time points following stroke, encompassing both sub-acute and chronic phases of recovery. Differences in neurological recovery, rehabilitation exposure, and adaptation processes across these stages may have influenced functional performance, fear of falling, and HRQoL. Although no significant subgroup differences were observed based on time since stroke in this exploratory analysis, future studies with larger samples and longitudinal designs should further investigate stage-specific recovery patterns. Finally, additional psychosocial and cognitive factors such as fear-avoidance beliefs, depression or cognitive impairment, were not assessed, even though they may influence fear of falling and participation after stroke. Future studies should incorporate these multidimensional contributors to better reflect the complexity of post-stroke recovery.

### 4.7. Implications for Clinical Practice

The findings of this study have clear clinical implications. The clinical implications outlined below are derived from the demonstrated associations among psychological, functional, and clinical outcomes observed in this cohort, highlighting the relevance of integrated assessment and intervention during inpatient post-stroke rehabilitation. The high prevalence of fear of falling and its strong links to mobility and HRQoL highlight the need for routine screening using tools such as the FES-I. Rehabilitation should integrate targeted balance training, gait re-education, and strength exercises, complemented by cognitive-behavioral strategies to address the psychological components of fear of falling. Identifying high-risk individuals—those with low TT scores, reduced independence, or elevated FES-I results—can help tailor rehabilitation pathways and fall-prevention efforts. The observed dissociation between high fear of falling and predominantly low objective fall risk further underscores the necessity of routinely assessing fear of falling independently from physical performance measures and incorporating tailored psychological and behavioral interventions alongside conventional balance and gait rehabilitation. Overall, the strong association between mobility, fear of falling, and HRQoL emphasizes the importance of holistic, interdisciplinary rehabilitation that supports both physical function and emotional wellbeing.

## 5. Conclusions

This exploratory study addressed three specific aims related to fear of falling in post-stroke inpatients undergoing rehabilitation. First, fear of falling was strongly associated with balance and gait performance, confirming that higher levels of fall-related concern are closely linked to poorer objective mobility outcomes. Second, fear of falling showed significant relationships with neurological deficit severity, functional independence, and health-related quality of life, indicating that fear of falling reflects a clinically meaningful, functionally driven psychological construct that extends beyond isolated mobility impairments. Third, fear of falling severity was not significantly influenced by demographic characteristics or stroke-related factors such as sex, stroke type, place of residence, or time since stroke, suggesting that fear of falling is pervasive across inpatient post-stroke populations rather than confined to specific subgroups. Specifically, the strong association between fear of falling and impaired mobility supports the recommendation to combine balance and gait training with targeted psychological strategies during rehabilitation. Future research should incorporate longitudinal designs and multidimensional interventions to better understand and modify these complex recovery pathways.

## Figures and Tables

**Table 1:** Demographic characteristics of the study group.

Variable	Category	N	%
Age	50–59 years	3	7.69
	60–69 years	14	35.90
	70–79 years	15	38.46
	≥80 years	7	17.95
Sex	Female	20	51.28
	Male	19	48.72
Marital status	Single	2	5.13
	Divorced	4	10.26
	Widowed	11	28.21
	Married	22	56.41
Place of residence	<50,000 inhabitants	14	35.90
	>50,000 inhabitants	14	35.90
	Rural area	11	28.21
Education level	Primary	1	2.56
	Secondary	34	87.18
	Higher	4	10.26

Notes: N—number of participants; %—percentage of participants.

**Table 2:** Clinical characteristics of the study group.

Variable	Category	N	%
Type of stroke	Hemorrhagic	11	28.21
	Ischemic	28	71.79
Stroke lateralization *	Left hemisphere	19	48.72
	Right hemisphere	26	66.67
Time since last stroke	<1 month	5	12.82
	1–3 months	17	43.59
	3–6 months	9	23.08
	>6 months	8	20.51
Number of strokes	1	32	82.05
	2	6	15.38
	3	1	2.56
	>3	0	0
Neurological deficits *	Right-sided paresis	18	46.15
	Left-sided paresis	21	53.85
	Speech disturbance	8	20.51
	Visual disturbance	5	12.82
	Balance/coordination problems	26	66.67
	Walking difficulties	29	74.36
	Cognitive deficits	3	7.69
	Hearing impairment	1	2.56
	None	1	2.56

Notes: Multiple responses possible, N—number of participants; %—percentage of participants. * Multiple-choice question.

**Table 3:** Fear of falling levels according to FES-I.

FES-I Category	Score Range	N	%
No or minimal fear	16–19	5	12.82%
Moderate fear	20–27	10	25.64%
High fear	28–64	24	61.54%

Notes: N—number of participants; %—percentage of participants. Abbreviations: FES-I—Falls Efficacy Scale-International.

**Table 4:** Fall risk according to Tinetti Test.

TT Risk Category	Score Range	N	%
High fall risk	0–18	7	17.95%
Moderate fall risk	19–23	7	17.95%
Low fall risk	24–28	25	64.10%

Notes: N—number of participants; %—percentage of participants. Abbreviations: TT—Tinetti Test.

**Table 5:** SF-36 summary scores.

Skala SF-36	Mean ± SD	Median (IQR)	Min–Max
Physical Functioning (PF)	63.9 ± 36.8	75 (30–100)	0–100
Role Physical (RP)	63.5 ± 41.2	100 (0–100)	0–100
Bodily Pain (BP)	68.6 ± 23.9	70 (60–80)	20–100
General Health (GH)	60.8 ± 22.7	60 (45–75)	25–100
Vitality (VT)	61.3 ± 24.6	60 (40–80)	0–100
Social Functioning (SF)	71.8 ± 25.9	80 (60–100)	0–100
Role Emotional (RE)	68.4 ± 39.5	100 (0–100)	0–100
Mental Health (MH)	65.2 ± 22.1	70 (50–80)	20–100
Total score	65.4 ± 18.9	66.9 (55.4–78.6)	33.4–93.1

Notes: M—mean, SD—standard deviation, Me—median, IQR—interquartile range, Min—minimal, Max—maximal. Abbreviations: SF-36—36-Item Short Form Health Survey.

**Table 6:** Comparisons by sex.

Variable	Women M ± SD	Men M ± SD	*p*-Value
FES-I	36.75 ± 15.96	35.11 ± 13.38	0.673
TT	15.15 ± 11.54	14.32 ± 8.25	0.725

Notes: Mann–Whitney U test. Abbreviations: FES-I—Falls Efficacy Scale-International, TT—Tinetti Test.

**Table 7:** Comparisons by stroke type.

Variable	Hemorrhagic M ± SD	Ischemic M ± SD	*p*-Value
FES-I	30.45 ± 13.90	38.11 ± 14.53	0.179
TT	19.09 ± 8.78	13.04 ± 10.01	0.121

Notes: Mann-Whitney U test. Abbreviations: FES-I—Falls Efficacy Scale-International, TT—Tinetti Test.

**Table 8:** Comparisons by place of residence.

Variable	Village M ± SD	Town <50 k M ± SD	City >50 k M ± SD	*p*-Value
FES-I	32.82 ± 13.31	35.93 ± 10.62	38.43 ± 18.94	0.681
TT	16.73 ± 8.36	15.43 ± 8.21	12.50 ± 12.62	0.605

Notes: Kruskal–Wallis + Dunn’s tests. Abbreviations: FES-I—Falls Efficacy Scale-International, TT—Tinetti Test.

**Table 9:** Comparisons by time since stroke.

Variable	<1 Month M ± SD	1–3 Months M ± SD	3–6 Months M ± SD	>6 Months M ± SD	*p*-Value	Dunn
FES-I	44.40 ± 22.20	32.88 ± 14.47	42.89 ± 12.56	29.38 ± 5.48	0.136	all ns
TT	10.00 ± 12.23	16.82 ± 10.02	9.11 ± 9.44	19.62 ± 5.13	0.080	all ns

Notes: Kruskal-Wallis + Dunn’s tests. Abbreviations: FES-I—Falls Efficacy Scale-International, TT—Tinetti Test, ns—non-significant.

**Table 10:** Correlations between SF-36 and key outcomes.

Variable	rs	*p*-Value
NIHSS	0.65	<0.001
BI	−0.71	<0.001
FES-I	0.76	<0.001
TT	−0.78	<0.001

Notes: rs—Spearman’s correlation coefficient. Abbreviations: SF-36—36-Item Short Form Health Survey, NIHSS—National Institutes of Health Stroke Scale, BI—Barthel Index, FES-I—Falls Efficacy Scale-International, TT—Tinetti Test.

## Data Availability

The data generated in this study will be included in the results of the published article. The data that support the findings of this study are available on request from the corresponding author (RD), upon reasonable request, as is the full protocol.

## Abbreviations

The following abbreviations are used in this manuscript:

BI	Barthel Index
BMI	Body Mass Index
FES-I	Falls Efficacy Scale-International
FoF	fear of falling
HRQoL	health-related quality of life
IRQ	interquartile ranges
NIHSS	National Institutes of Health Stroke Scale
SF-36	36-Item Short Form Health Survey
TT	Tinetti Test

## References

[1] Feigin V.L., Brainin M., Norrving B., Martins S.O., Pandian J., Lindsay P., Grupper M.F., Rautalin I. (2025). World Stroke Organization: Global Stroke Fact Sheet 2025. Int. J. Stroke.

[2] Tahiraj A., König H.-H., Hajek A. (2024). Experiencing Cerebrovascular Diseases like Stroke and Fear of Falling: Longitudinal Results from the Survey of Health, Ageing and Retirement in Europe. Geriatrics.

[3] Larén A., Odqvist A., Hansson P.-O., Persson C.U. (2018). Fear of Falling in Acute Stroke: The Fall Study of Gothenburg (FallsGOT). Top. Stroke Rehabil..

[4] Schinkel-Ivy A., Inness E.L., Mansfield A. (2016). Relationships between Fear of Falling, Balance Confidence, and Control of Balance, Gait, and Reactive Stepping in Individuals with Sub-Acute Stroke. Gait Posture.

[5] Liu T.-W., Ng S.S., Kwong P.W., Ng G.Y. (2015). Fear Avoidance Behavior, Not Walking Endurance, Predicts the Community Reintegration of Community-Dwelling Stroke Survivors. Arch. Phys. Med. Rehabil..

[6] Kongsuk J., Brown D.A., Hurt C.P. (2019). Dynamic Stability during Increased Walking Speeds Is Related to Balance Confidence of Older Adults: A Pilot Study. Gait Posture.

[7] Komalasari R., Mpofu E., Chen H., Talluntondok E.B., Uligraff D.K., Zhan R., Thiamwong L. (2024). Higher Dynamic Balance Performance Was Associated With Cognitive Function Among U.S. Community-Dwelling Low-Income Older Adults. SAGE Open Nurs..

[8] Papalia G.F., Papalia R., Diaz Balzani L.A., Torre G., Zampogna B., Vasta S., Fossati C., Alifano A.M., Denaro V. (2020). The Effects of Physical Exercise on Balance and Prevention of Falls in Older People: A Systematic Review and Meta-Analysis. J. Clin. Med..

[9] Schoene D., Heller C., Aung Y.N., Sieber C.C., Kemmler W., Freiberger E. (2019). A Systematic Review on the Influence of Fear of Falling on Quality of Life in Older People: Is There a Role for Falls?. Clin. Interv. Aging.

[10] Xie Q., Pei J., Gou L., Zhang Y., Zhong J., Su Y., Wang X., Ma L., Dou X. (2022). Risk Factors for Fear of Falling in Stroke Patients: A Systematic Review and Meta-Analysis. BMJ Open.

[11] Bąk E., Młynarska A., Marcisz C., Kadłubowska M., Marcisz-Dyla E., Sternal D., Młynarski R., Krzemińska S. (2022). Kinesiophobia in Elderly Polish Patients After Ischemic Stroke, Including Frailty Syndrome. Neuropsychiatr. Dis. Treat..

[12] Park J., Yoo I. (2014). Relationships of Stroke Patients’ Gait Parameters with Fear of Falling. J. Phys. Ther. Sci..

[13] Schmid A.A., Van Puymbroeck M., Knies K., Spangler-Morris C., Watts K., Damush T., Williams L.S. (2011). Fear of Falling among People Who Have Sustained a Stroke: A 6-Month Longitudinal Pilot Study. Am. J. Occup. Ther..

[14] von Elm E., Altman D.G., Egger M., Pocock S.J., Gøtzsche P.C., Vandenbroucke J.P., STROBE Initiative (2007). Strengthening the Reporting of Observational Studies in Epidemiology (STROBE) Statement: Guidelines for Reporting Observational Studies. BMJ.

[15] Wiśniewski A., Filipska K., Puchowska M., Piec K., Jaskólski F., Ślusarz R. (2021). Validation of a Polish Version of the National Institutes of Health Stroke Scale: Do Moderate Psychometric Properties Affect Its Clinical Utility?. PLoS ONE.

[16] Kuźmicz I., Brzostek T., Górkiewicz M. (2008). Barthel Questionnaire As Measurement Tool For Physical Independence of Older Adults. Med. Stud..

[17] Faria-Fortini I., Polese J.C., Faria C.D.C.M., Scianni A.A., Nascimento L.R., Teixeira-Salmela L.F. (2021). Fall Efficacy Scale-International Cut-off Score Discriminates Fallers and Non-Fallers Individuals Who Have Had Stroke. J. Bodyw. Mov. Ther..

[18] Zak M., Makara-Studzińska M., Mesterhazy A., Mesterhazy J., Jagielski P., Januszko-Szakiel A., Sikorski T., Jaworski P., Miszczuk R., Brola W. (2022). Validation of FES-I and Short FES-I Scales in the Polish Setting as the Research Tools of Choice to Identify the Fear of Falling in Older Adults. Int. J. Environ. Res. Public Health.

[19] Colombo P., Taveggia G., Chiesa D., Penati R., Tiboni M., De Armas L., Casale R. (2019). Lower Tinetti Scores Can Support an Early Diagnosis of Spatial Neglect in Post-Stroke Patients. Eur. J. Phys. Rehabil. Med..

[20] Zak M., Krupnik S., Puzio G., Staszczak-Gawelda I., Czesak J. (2015). Assessment of Functional Capability and On-Going Falls-Risk in Older Institutionalized People after Total Hip Arthroplasty for Femoral Neck Fractures. Arch. Gerontol. Geriatr..

[21] Naczk M., Marszalek S., Naczk A. (2020). Inertial Training Improves Strength, Balance, and Gait Speed in Elderly Nursing Home Residents. Clin. Interv. Aging.

[22] Babiuch A.S., Oestervemb K., Lipińska A., Stańczak M.L., Cholewa M., Makulec K., Nowakowska K., Derengowska M.H. (2021). Differences in the Level of Physical Fitness and Mobility among Older Women with Osteoporosis and Healthy Women-Cross-Sectional Study. Sci. Rep..

[23] Braga M.A.F., Faria-Fortini I., Soares C.L.d.A., Rodrigues N.A.G., Sant Anna R.V., Faria C.D.C.d.M. (2024). Acute Clinical Outcomes Predict Both Generic and Specific Health-Related Quality of Life Six and 12 Months after Stroke: A One-Year Prospective Study Developed in a Middle-Income Country. J. Stroke Cerebrovasc. Dis..

[24] Kłosiński M., Tomaszewski K.A., Tomaszewska I.M., Kłosiński P., Skrzat J., Walocha J.A. (2014). Validation of the Polish Language Version of the SF-36 Health Survey in Patients Suffering from Lumbar Spinal Stenosis. Ann. Agric. Environ. Med..

[25] Canbek J., Fulk G., Nof L., Echternach J. (2013). Test-Retest Reliability and Construct Validity of the Tinetti Performance-Oriented Mobility Assessment in People with Stroke. J. Neurol. Phys. Ther..

[26] Schmid A.A., Van Puymbroeck M., Altenburger P.A., Dierks T.A., Miller K.K., Damush T.M., Williams L.S. (2012). Balance and Balance Self-Efficacy Are Associated with Activity and Participation after Stroke: A Cross-Sectional Study in People with Chronic Stroke. Arch. Phys. Med. Rehabil..

[27] Pin T.W., Winser S.J., Chan W.L.S., Chau B., Ng S., Wong T., Mak M., Pang M. (2024). Association between Fear of Falling and Falls Following Acute and Chronic Stroke: A Systematic Review with Meta-Analysis. J. Rehabil. Med..

[28] Chen Y., Du H., Song M., Liu T., Ge P., Xu Y., Pi H. (2023). Relationship between Fear of Falling and Fall Risk among Older Patients with Stroke: A Structural Equation Modeling. BMC Geriatr..

[29] Schmid A.A., Arnold S.E., Jones V.A., Jane Ritter M., Sapp S.A., Van Puymbroeck M. (2015). Fear of Falling in People With Chronic Stroke. Am. J. Occup. Ther..

[30] Goh H.-T., Nadarajah M., Hamzah N.B., Varadan P., Tan M.P. (2016). Falls and Fear of Falling After Stroke: A Case-Control Study. PM R.

[31] Hussain N., Hansson P.-O., Persson C.U. (2021). Prediction of Fear of Falling at 6 Months after Stroke Based on 279 Individuals from the Fall Study of Gothenburg. Sci. Rep..

[32] Yang Y., Li M., Ding X., Zhang L., Jin M., Jin Y. (2025). Classification and Influencing Factors of Fear of Falling in Elderly Stroke Patients: A Latent Profile Analysis. Aging Clin. Exp. Res..

[33] Tian X., Mai Y.-H., Guo Z.-J., Chen J.-W., Zhou L.-J. (2024). Contributing Factors and Interventions for Fear of Falling in Stroke Survivors: A Systematic Review. Top. Stroke Rehabil..

[34] Djurovic O., Mihaljevic O., Radovanovic S., Kostic S., Vukicevic M., Brkic B.G., Stankovic S., Radulovic D., Vukomanovic I.S., Radevic S.R. (2021). Risk Factors Related to Falling in Patients after Stroke. Iran. J. Public Health.

[35] Lee E.S., Kim B. (2024). The Impact of Fear of Falling on Health-Related Quality of Life in Community-Dwelling Older Adults: Mediating Effects of Depression and Moderated Mediation Effects of Physical Activity. BMC Public Health.

[36] Chiu C.Y., Ng M.Y.-H., Lam S.C., Hui K.Y., Keung C.H., Ouyang H., Li X., Pang M.Y.-C. (2023). Effect of Physical Exercise on Fear of Falling in Patients with Stroke: A Systematic Review and Meta-Analysis. Clin. Rehabil..

[37] Shen J., Gu X., Yao Y., Li L., Shi M., Li H., Sun Y., Bai H., Li Y., Fu J. (2023). Effects of Virtual Reality-Based Exercise on Balance in Patients With Stroke: A Systematic Review and Meta-Analysis. Am. J. Phys. Med. Rehabil..

[38] Kunkel D., Mamode L., Burnett M., Pickering R., Bader D., Donovan-Hall M., Cole M., Ashburn A., Bowen C. (2023). Footwear Characteristics and Foot Problems in Community Dwelling People with Stroke: A Cross-Sectional Observational Study. Disabil. Rehabil..

[39] Cudejko T., Akpan A., D’Août K. (2025). Biomechanical Mechanisms Underlying the Effect of Minimalist Footwear on Walking Stability in Persons with a History of Falls. Commun. Med..

[40] Branthwaite H., Chockalingam N. (2019). Everyday Footwear: An Overview of What We Know and What We Should Know on Ill-Fitting Footwear and Associated Pain and Pathology. Foot.

